# Impact of interfering substances on the bactericidal efficacy of different commercially available hypochlorous acid-based wound irrigation solutions commonly found in South-East Asia

**DOI:** 10.1017/ash.2025.81

**Published:** 2025-09-03

**Authors:** Jiann Wen Yap, Neni Iffanida Ismail, Cheng Shoou Lee, Ding Yuan Oh

**Affiliations:** 1 Queen Elizabeth Hospital, Sabah, Malaysia; 2 TECOLAB SDN BHD, Kuala Lumpur, Malaysia; 3 Schülke & Mayr (Asia) Pte Ltd, Singapore

## Abstract

**Objectives:** Chronic wounds are commonly manifested with biofilms that result in delayed healing and recurring infection episodes. Due to increasing antimicrobial resistance, antiseptics have played a significant role in wound infection management. To date, there is no standardised method to assess the ‘true’ antimicrobial efficacy of different antimicrobials, especially in testing condition that stipulate a high protein environment in the wound. As clinicians often rely on the antimicrobial efficacy profiles for product selection, a rigorous testing is warranted. Hypochlorous acid (HOCl)-based solutions have been introduced as a good alternative for wound cleansing but the assessment of its antimicrobial activities in a stipulate wound environment is limited. In this study, we assessed the *in vitro* bactericidal activities of 7 commercially available wound irrigation products commonly found in South-East Asia. **Method:** The evaluation was conducted using quantitative suspension method, EN 13727 in either low or high protein conditions. **Results:** Under low protein conditions, four out of the five HOCl products achieved bactericidal activity (≥ 5 ^log^10 reduction factor; RF) within 2 to 5 minutes, and only one product achieved 5 log RF at 15 seconds. None of the HOCl achieved 5 log RF under high protein, even after 30 minutes of exposure time. In contrast, protein interference on the antimicrobial activities of polyhexamethylene biguanide-based product is less pronounced (low protein: 60 seconds vs. high protein: 2 minutes to attain ≥ 5 log RF). Octenidine dihydrochloride is the only active not affected by protein interference achieving ≥ 5 log10 RF within 15 seconds in both low and high protein conditions. **Conclusion:** These findings warrant the need to screen antimicrobial wound care products, especially HOCl-based products, in high protein condition to better reflect the antimicrobial activities in wound care.

